# Leader-Inspired Nutrition: A Framework for Promoting Healthy Nutrition Behaviors and a Nutritionally Fueled and Fit Military Force

**DOI:** 10.3390/nu17243835

**Published:** 2025-12-08

**Authors:** Tanisha L. Currie, Cindy Crawford, Patricia A. Deuster, Andrea T. Lindsey, Melissa Rittenhouse, Katie Kirkpatrick, Deborah Robinson, Melissa R. Troncoso, Mary McCarthy, Courtney Paolicelli, Mamusu Turay, Maria McConville, Shellye Suttles, Matthew P. Rabbitt, Amy B. Adler, Jonathan M. Scott

**Affiliations:** 1 Center for Enabling Capabilities, Walter Reed Army Institute of Research, Fort Glen Annex, Silver Spring, MD 20910, USA; 2 Consortium for Health and Military Performance, Department of Military and Emergency Medicine, F. Edward Hébert School of Medicine, Uniformed Services University, Bethesda, MD 20814, USA; cindy.crawford.ctr@usuhs.edu (C.C.); pdeuster@me.com (P.A.D.); andrea.lindsey.ctr@usuhs.edu (A.T.L.); melissa.rittenhouse.ctr@usuhs.edu (M.R.); katie.kirkpatrick.ctr@usuhs.edu (K.K.); debrobinson19@cox.net (D.R.); maria.mcconville.ctr@usuhs.edu (M.M.); jonathan.scott@usuhs.edu (J.M.S.); 3 Henry M. Jackson Foundation for the Advancement of Military Medicine, Inc., Bethesda, MD 20817, USA; 4 Daniel K. Inouye Graduate School of Nursing, Uniformed Services University, Bethesda, MD 20814, USA; melissa.troncoso@usuhs.edu; 5 Madigan Army Medical Center, Tacoma, WA 98431, USA; mary.s.mccarthy1.civ@health.mil; 6 Office of Military Community and Family Policy, Department of Defense, Alexandria, VA 22350, USA; courtney.p.paolicelli.civ@mail.mil; 7 Acquisition and Sustainment, Department of Defense, Washington, DC 20301, USA; mamusu.turay.ctr@mail.mil; 8 Economic Research Service, U.S. Department of Agriculture, Kansas City, MO 64105, USA; shellye.suttles@usda.gov (S.S.); matthew.rabbitt@usda.gov (M.P.R.); 9 Center for Military Psychiatry and Neuroscience, Walter Reed Army Institute of Research, Silver Spring, MD 20910, USA; amy.b.adler.civ@health.mil

**Keywords:** food insecurity, Leader-Inspired Nutrition, leadership, military nutrition environment, nutrition, nutrition knowledge, obesity, policy, Total Force Fitness, wellness resources

## Abstract

As role models, military leaders are in a unique position to influence and inspire military members. Military leaders identify and address challenges that threaten operational readiness. A growing national concern across all military branches of service is Service members’ suboptimal nutritional fitness, underpinned by increasing rates of food insecurity and obesity, inadequate nutrition knowledge and cooking skills, and limited access to healthier food options within the military nutrition environment. Military leaders set the standards, conditions, and policies for achieving their organization’s goals. Therefore, Leader-Inspired Nutrition (LIN) is an evidence-based, proposed framework for how military leaders can engage Service members within the domain of nutritional fitness. LIN builds upon the nutritional fitness domain of the Department of Defense’s (DoD’s) Total Force Fitness framework for optimal performance. Subject matter experts in areas of nutrition science and education, military food environment, and leadership from DoD and other outside federal agencies were selected and asked to identify evidence-based strategies leaders can use to optimize nutrition readiness. This concept led to the development of the seven pillars of the LIN framework, outlining how it can be operationalized for leaders to inspire a more nutritionally fit DoD fighting force.

## 1. Introduction

Nutrition holds profound significance in shaping human health, well-being, and physical performance across all stages of life [1,2,3,4,5,6]. Nutrition also plays a role in work performance [7,8,9]. Nowhere is performance perhaps more important than in high-stakes occupations like the military. Nutrition is intricately linked to national security; without consistent availability of healthy food choices and Service members (SMs) engaging in positive nutritional choices, the military may not be adequately prepared to operate at peak performance and maintain optimal health. Therefore, this article will outline how military leaders can engage with SMs on nutrition by leveraging an innovative, evidence-based framework entitled Leader-Inspired Nutrition (LIN). Prior to this discussion, the article will detail threats to readiness within the U.S. military that are directly related to the food environment and poor nutritional status in America.

One nutrition-related threat to military readiness is food insecurity. Food insecurity is defined by “the limited or uncertain availability of nutritionally adequate or safe foods or limited or uncertain ability to acquire acceptable foods in socially acceptable ways” [10]. Behaviors associated with food insecurity include being unable to afford balanced meals, having to decrease the serving size, or simply having to go hungry. Individuals considered food-insecure are at risk for adverse physical and mental health outcomes [11,12]. For military members, in addition to these adverse outcomes and behaviors [11], food insecurity is linked to occupational stressors that can affect military readiness [11,13,14]. According to Rabbitt et al. (2024), the prevalence of food insecurity was higher in the military population (25.3%) compared to the civilian adult population (10.1%) in 2018 and 2020 [15]. The higher risk for food insecurity among SMs remains even after adjusting for sociodemographic factors. This issue is concerning given the association between food insecurity, mental health, and intentions to depart military service. According to Beymer et al. (2021), of 5677 soldiers surveyed at one U.S. Army installation, 52% expressed their intention to depart from the Army after their current service period, citing outcomes associated with anxiety, depression, and thoughts of suicide [16]. Food insecurity was significantly and independently associated with the observed mental health concerns expressed by SMs in this study.

A second nutrition-related threat to military readiness is obesity. Obesity is a major public health issue because the U.S. military only accepts healthy and physically fit recruits. The increasing prevalence of obesity in society presents a growing obstacle to the military’s ability to meet its recruitment goals and objectives [17]. Over 70% of young adults (17–24 years of age) are ineligible to serve in the military, with obesity being the number one reason. In addition, the prevalence of soldiers who are obese (body mass index > 30) has increased from 17% to 20% in five years, spanning from 2017 to 2021 [18]. This severely impacts future SMs as 90% of military applicants are aged 17 to 24 years old. All branches of service have recruiting challenges, partially due to an overweight/obese and unfit pool of potential recruits. Obesity is also linked to food security issues when individuals are unable to access healthy foods and rely on the consumption of energy-dense and processed foods instead. Furthermore, being obese is a risk factor for many chronic health conditions, such as musculoskeletal injuries that result in lost duty time [19,20,21,22,23].

Poor dietary intake is also a threat to military readiness [24]. Specifically, a lack of fruit and vegetable consumption has been associated with the rising rates of obesity in the military [25]. According to a 2019 Centers for Disease Control and Prevention report, 12.3% and 10.0% of surveyed adults met fruit and vegetable intake recommendations, respectively [26]. The latest Health of the Force reports that less than 40% of soldiers met the minimum nutrition targets of eating at least two servings of fruit per day (27%) or at least two servings of vegetables per day (38%) [18].

What leads to these high levels of food insecurity and obesity, and low levels of fruit and vegetable intake? Contributing factors might include a lack of nutrition (or cooking) knowledge or not knowing where to obtain credible nutrition information; lack of physical access to healthy foods, such as for those living in communities or barracks on a military installation with limited food access and without transportation (commonly referred to as food deserts), or too many non-nutritious food and beverage choices in the work, home, and community environment (e.g., fast food, grab and go, vending machines); lack of positive nutritional behaviors; and financial insecurity. These factors can all have a critical impact on the purchasing and consumption of nutritious foods [25].

In light of the importance of nutrition to military performance and health, it is important to consider ways to counteract food insecurity, obesity, and poor dietary intake. One way to address these gaps in nutrition is to leverage the role of military leaders. In the military environment, leaders play a vital role in influencing the motivation and performance of their subordinates [27,28,29]. Moreover, research has found that when leaders engage in behaviors that promote certain health-related outcomes, their unit members are more likely to report specific healthy outcomes [30]. 

### 1.1. The Road from Readiness Frameworks to Leader-Inspired Nutrition as an Innovative Approach

Total Force Fitness (TFF) was adopted by the Department of Defense (DoD) in 2009 as a holistic health framework for military fitness and readiness. TFF encompasses the physical, social, psychological, environmental, financial, spiritual, medical and dental, and nutritional health and fitness aspects of well-being and enables individuals to get healthy and achieve optimal performance. Within TFF, nutritional fitness means having access to and choosing foods known to fuel performance, support mission readiness, and sustain optimal health [31]. Nutritional fitness is an essential component to ensure TFF of SMs and directly affects readiness, resilience, and retention, which are imperative for our national defense. Stemming from the TFF framework came readiness initiatives, such as The Army’s Holistic Health and Fitness (H2F) initiative, which equips soldiers with personnel and the resources to take charge of their health, fitness, and well-being, as well as injury and disease prevention. Unlike TFF, which is composed of 8 domains of health, H2F trimmed its model to 5 domains, which consist of physical, nutritional, mental, spiritual, and sleep readiness [32].

Leader-Inspired Nutrition (LIN) is a conceptual framework that builds upon these readiness frameworks and initiatives. LIN focuses on a leader’s role in building a culture of health by promoting healthy nutrition behaviors within the organization [33]. LIN leverages tools for engaging and empowering leaders to create a healthy workplace environment through a variety of strategies that impact SM nutrition. Military leaders at all levels are uniquely positioned to mitigate unhealthy nutrition practices and encourage better ones in various ways. If leaders practice and prioritize healthy lifestyle habits, they will impact individuals, units, and organizations, cultivating a culture of health in their respective areas. Additionally, it is essential to acknowledge that leaders come from diverse backgrounds and hold various health beliefs; as such, SMs must receive unified messaging and access to credible resources regarding nutrition readiness.

### 1.2. Leader-Inspired Nutrition Principles

LIN was initially developed by a core group of scientists, healthcare practitioners, military leaders, and other key stakeholders with knowledge of nutrition and its effect on SM health and fitness. Subject matter experts (SMEs) were recruited for specific pillars of the LIN framework and received a video on LIN’s goals, definition, and conceptual framework. Twelve SMEs agreed to contribute. They were first interviewed, then asked to provide a white paper addressing two core questions: (a) How can leaders inspire employees’ nutrition for increased performance and productivity? and (b) How can leaders provide constructive feedback and support to employees struggling with nutrition or overall wellness? The core group of SMEs who conceptualized LIN reviewed all white papers and collated the findings to build and refine the LIN framework.

LIN follows five principles that leaders can apply in their units: (1) focus on the whole health and well-being of the SM, (2) create opportunities to support nutrition readiness and healthy behaviors by getting to know unit members’ and demonstrating how they value unit health and performance, (3) learn what factors influence unit members’ eating behaviors and food choices, (4) facilitate positive changes and policies in the military nutrition environment, and (5) recognize nutrition readiness as a link to psychological, social, and physical health, military readiness, and quality of life [33].

The framework of LIN outlines specific core elements that are important to the military nutrition environment and leadership context. LIN, as a nutrition leadership framework, provides “what leaders can do” to engage in nutrition readiness; and the principles of LIN provide “how” this can be achieved through focus on well-being, military nutrition readiness and environment, and factors impacting SM eating behaviors.

## 2. The Pillars of Leader-Inspired Nutrition

Seven pillars, based on nutritional readiness threats and evidence-based resources, were created to promote a culture of health. These pillars are as follows: integrate nutrition 101; promote a performance-focused environment; integrate dietary supplements 101; model top-down nutrition behaviors; consider economic factors that affect nutrition choices; promote utilization of DoD and partners’ wellness resources; and evaluate TFF impact.

**Figure 1:**
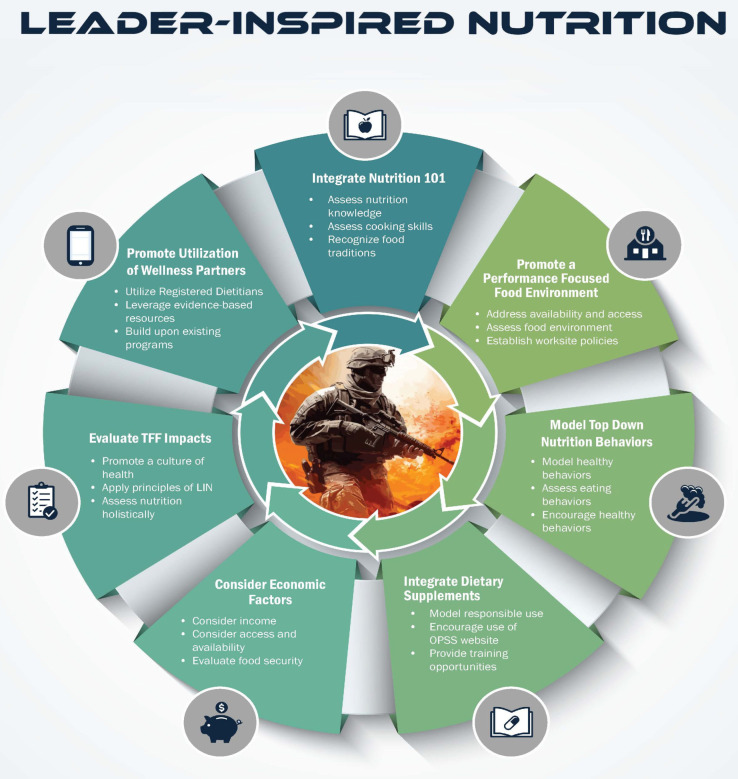
Leader-Inspired Nutrition Framework. Note: LIN is Leader-Inspired Nutrition; OPSS is Operation Supplement Safety; and TFF is Total Force Fitness.

This article details the previously mentioned pillars, except the pillar of integrating dietary supplements knowledge, as it is detailed in a separate perspective that accompanies this article [34]. The concept of Leader-Inspired Nutrition is depicted in Figure 1 below, an image that demonstrates the seven pillars and how leaders can engage with their unit members.

### 2.1. Pillar 1: Integrate Nutrition 101

Knowledge is a fundamental construct that is critical to the performance of any health-related behavior. As posited in social cognitive theory, knowledge and skill are needed to successfully perform a health-promoting behavior [35]. As such, it is important to begin any health and nutrition intervention by first gauging nutrition knowledge across a range of topics. Currently, there is no universal Nutrition 101 curriculum implemented across an SM’s career. While leaders can implement tools to assess nutrition knowledge, a Nutrition 101 course for all SMs would be an efficient way to ensure a common baseline understanding. This lack of training remains a gap for the DoD.

Additionally, nutritional fitness is not a one-size-fits-all solution, meaning optimal nutrition must be achieved in a way that is appropriate and personalized to the individual SM with a focus on their priorities and needs. In fact, the 2020–2025 Dietary Guidelines for Americans encourages Americans to “customize and enjoy nutrient-dense food and beverage choices to reflect personal preferences, cultural traditions, and budgetary considerations” [36]. These federal guidelines influence military nutrition by providing the foundation for menu planning and standards [37]. When integrating nutrition 101, it is imperative to recognize and appreciate the various food traditions, preferences, and habits of SMs. Although leaders are not expected to be nutrition experts, they should seek out SME support from their installation [38,39,40,41].

#### 2.1.1. Assess Nutrition Knowledge

To begin, leaders can get smart about healthy food choices and foster an environment that encourages healthy habits by informally assessing SM’s current nutrition knowledge, skills, and abilities. In fact, a study conducted by Sheafer et al. showed that nutrition knowledge was positively associated with improved eating behaviors and diet quality [42]. Some easy ways to assess nutritional knowledge are by observing physical readiness (i.e., physical fitness tests), meals, and daily life choices. A leader should note poor performance (i.e., inability to finish a task because of fatigue, not meeting fitness standards, lack of a fit military appearance) among their personnel; these are red flags, and may indicate lack of sleep, poor nutrition, or a medical issue. Increased education and an appointment with a registered dietitian (RD) might help military members prevent and/or treat such issues.

After observing areas of opportunity, leaders can provide resources and a space to improve self-efficacy through modeling and encouraging mindful dialog about healthful behaviors. Leaders can use a top-down approach by role modeling healthy nutrition choices. In addition, the leader can start a conversation to gauge interest and readiness for change [43] (i.e., stages of change include precontemplation, contemplation, preparation, action, maintenance, and termination) to adapt healthier eating habits. Importantly, leaders should let an SM know they care about the SM’s success; a leader’s expression of caring may be enough for SMs to be motivated to take steps towards healthier habits. If SMs are not ready to make a change or are struggling to do so, leaders can determine what factors are inhibiting SMs from meeting their goals and successfully maintaining proper weight standards. Common factors might include a lack of nutrition knowledge, limited cooking skills, misconceptions, and attitudes such as “eating healthy tastes bad” or “preparing healthy food takes too long”.

In conducting a conversation, leaders could use open-ended questions, such as the ones below, to understand the variety of factors that influence nutrition choices and eating habits and encourage SMs to think about the choices they are making. Please note that the question sets listed below each respective section are intended to be illustrative and exploratory to serve as a guide for leaders. The SMEs developed illustrative and exploratory questions leaders can consider in how they approach their SM on these topics. These questions can be asked in group or individual settings, where appropriate, and can encourage better hydration and nutrition status of SMs. Additionally, leaders should exercise caution so as not to judge SMs for their eating habits but instead encourage and promote a healthy food environment that also appreciates balance.

Open-ended questions:

- Why did they skip lunch or eat a fried meal instead of a non-fried meal?
- What are their goals and how can nutrition help accomplish their goals? These questions will give some insight as to their nutrition knowledge.
- Are they able to meet their goals with the foods provided? If not, why not? It is important to determine the barriers to success.
- Look for common themes and assess the nutrition environment. Are there options they want to eat? Are there a variety of options? Do they want to eat in the military dining facility? If not, why not? If yes, are there nutritious options (whole grains, fresh fruits and vegetables, lean proteins)? Does the food look appealing? Is it served at correct temperatures and following food safety protocols? Are the portion sizes large enough to meet the caloric demands of the service member or are they still hungry when they leave? These are all key components to food acceptability.
- Do they eat out often or buy premade meals? Why are they choosing the specific meal options? Do they like what they are choosing? These questions will help determine whether changes are needed at the military dining facility and other venues to better serve the community.

With minimal time investment by leaders, it can be easy to assess the big picture among their personnel. In addition, leaders can assess the basics, such as where SMs are typically eating their meals and how they fill their plates, by taking a quick scan while members are eating at the dining facility. Learning more about the current landscape can help identify where leaders can assist and support their team.

Leaders should consider whether:

- SMs are balancing their plates with portions of fruit, vegetables, lean protein, and whole grains;
- SMs who do not eat at the DFAC balance their plates with portions of fruit, vegetables, lean protein, and whole grains;
- SMs appear to be enjoying the food and the environment, and what options are offered;
- SMs have adequate time to consume their food.

A future recommendation would be for all Services to offer nutrition education training. For example, a short tutorial or coursework could be created to explain the six essential nutrients (proteins, carbohydrates, fats, vitamins, minerals, and water), how these nutrients affect bodily functions, and how a lack of these nutrients can cause disorders, diseases, or suboptimal performance. Additionally, foundational nutrition courses can be created and implemented within military training courses throughout an SM’s career trajectory in an effort to consistently build on their foundational nutrition knowledge. Future trainers of these nutrition blocks should motivate SMs to critically think about their food options, how they fuel their bodies, when fueling occurs, and how their daily food habits can lead to optimized health or a threat to health readiness. For SMs who are eager to learn more now, leaders can refer them to the Warfighter Nutrition Guide for more information [39] or an RD for a personalized approach that optimizes their specific nutrition goals.

#### 2.1.2. Assess Cooking Skills

##### Self-Efficacy Toward Cooking

SMs might want or need to cook their own food due to factors such as missing open dining hours, shift work, and lack of access [25]. However, it is important to recognize that not every SM entering or currently serving in the military has the same basic levels of cooking knowledge. Just as with nutrition knowledge, it is important to assess cooking ability and self-efficacy in cooking, as these can be a major barrier to preparing meals. All in all, leaders should be aware of some of the food preparation barriers to SMs eating healthy foods so that they can properly connect the SM to the unit’s RD or other resources.

Some illustrative and exploratory questions for SM self-reflection may include:

- Do you know how to prepare your own meals?
- Can you make balanced, nutritious meals (lean protein, complex carbohydrates, vegetables)?
- Are there certain barriers in being able to prepare your own meal?
- Can you make a meal out of the food in your refrigerator or pantry, or food available at the commissary or local grocery store?
- Do you feel comfortable following a recipe?
- How comfortable are you cooking with basic appliances and ingredients, such as boiling water to make pasta, making a sandwich, stir-frying vegetables, baking fish in the oven, or making a salad?

There are educational opportunities where RDs, public health, Armed Forces Wellness Center staff, or other professional staff can offer to teach basic skills and assist in building confidence in cooking knowledge among SMs. For example, the military community has explored the incorporation of food choice selection and cooking as an educational experience for SMs. Teaching kitchens, such as the Total Force Kitchen (TFK), aim to improve SM diet quality through a culinary experience that encompasses principles of performance-based nutrition, physical fitness, and mindfulness. In a pilot study conducted on TFK, participants reported positive lifestyle changes in food quality selections and food preparation. Also, another key implication mentioned in the study was the potential impact on community empowerment through optimizing cooking in both the military and nonmilitary populations [44].

##### Assess Attitude Towards Healthy Foods

Different perceptions may play a role in food choices [45]; some common ones are that healthy foods are not aesthetically appealing or tasty, they cost more to make or buy, and they take too long to make. If misconceptions or concerns are present, leaders can role model healthy eating behaviors, share a meal prepared with healthy foods, and also include an RD in the discussion. Additionally, leaders can invite an RD to the unit’s table for a meal and a discussion on food perceptions.

##### Assess Knowledge of Cooking Skills

There are times when dining facilities will be closed, and SMs are on their own for a meal. For those who do not have the resources, equipment, or knowledge to cook, the only answer may be purchasing prepared food. Leaders who learn more about SM’s baseline cooking skills can help direct SMs to the right resources. Leaders can invite RDs to their respective units to assist in assessing SM cooking knowledge and skills. Informing the SM of the resources available to them would be helpful in this scenario. Some cooking classes may be available on installations. They may be offered by an RD, Armed Forces Wellness Centers, an exchange, or a commissary. If SMs are interested in learning and there are minimal resources available, this opportunity would be an optimal time for leaders to step in and put the resources together to share. For those interested, the Cooking Guide for Soldiers is a great resource [38].

### 2.2. Pillar 2: Promote a Performance-Focused Food Environment

The military nutritional environment (MNE) includes all food, beverages, and dietary supplement options available within military settings. A supportive MNE improves access to, availability of, and knowledge of high-performance, nutritious choices [41]. Leaders at all levels can have a powerful impact on nutritional fitness by assessing and addressing the food environment through the methods described below. A leader’s role in terms of promoting safe dietary supplement access, availability, and knowledge is also critical, but a leader should always promote nutritious food access and availability over dietary supplements. Dietary supplement education is a core pillar for LIN, which is detailed in a separate article [34].

The White House’s National Strategy on Hunger, Nutrition, and Health (2022) prioritized environments that “enable all people to easily make informed healthy choices, increase access to healthy food, encourage healthy workplace and school policies….” [46]. Research shows that when leaders build and model a culture of health and wellness, it encourages optimal health, performance, and productivity [47]. Leaders can start by modeling behaviors and incorporating healthy habits, such as allowing ample time for lunch or dinner meals, and supporting hydration and snack breaks, in their daily work environments. This modeling not only promotes healthy eating habits but also allows time for building trusting relationships, camaraderie, community engagement, and partaking in celebrations.

#### 2.2.1. Address Food Availability and Access

Convenient, tasty, and budget-friendly foods and beverages help fuel the military community where they live, work, and train. A supportive food environment makes it easier for the military community to select choices that positively impact their nutrition and overall wellness. As a first step, leaders can determine the supportiveness of their local nutrition landscape. The DoD’s standardized Military Nutrition Environment Assessment Tool (mNEAT) objectively evaluates a local installation or ship’s performance-focused food policies, access, and availability to identify areas of success and opportunities for improvement [48]. Leaders can endorse the next step, which involves implementing actionable change.

The mNEAT pilot research showed that leadership support, collaboration, and local control are facilitators of successful food environment assessment and intervention efforts [49]. Conversely, variable leadership support, insufficient awareness of mNEAT and its potential benefits, and limited resources for execution were noted as barriers [49]. At this time, no uniform policies exist to conduct, share, and report mNEAT results, either across the DoD or within a branch.

#### 2.2.2. Build Performance-Focused Environments Through Policies, Resources, and Experts

One of the most impactful tools to transform a performance-focused food environment is through establishing meaningful and specific healthy worksite policies or defining guidelines about a leader’s intent for health, wellness, and nutrition. Leaders can advocate for better access to and availability of healthier foods that positively impact performance and productivity through multiple channels (e.g., in-person meetings, print, social media, briefs).

Leaders can also support educational opportunities and the inclusion of food environment optimization in performance and fitness initiatives. A strategic plan can help amplify consistent messaging about the important role the local food environment plays in performance. Leaders can leverage existing programs such as Go for Green^®^/Fueled to Fight^®^ to facilitate the selection of performance-focused choices at military food service operations [50].

Available expert assets, such as RDs, health promotion coordinators, and health or wellness councils or work groups, can contribute knowledge, offer resources, assist with the implementation of LIN, evaluate return on investment, and synchronize efforts to be part of community-wide targets. Hence, these policies help facilitate nutrition initiatives, pilots, and programs by providing official justification and support. All levels of leadership can help amplify performance and wellness policies, resources, and experts to transform their local food environment.

### 2.3. Pillar 3: Model Top-Down Nutrition Behaviors

For LIN to be successful, the modeling of top-down nutrition behaviors needs to be integral to all pillars of LIN, which is built through engaged leadership, community, trust, and role modeling. Leaders set the example; if leaders practice healthy nutrition practices, SMs are more likely to do the same. Empowered, health-conscious leaders motivate SMs toward positive nutritional behaviors [51]. SMs can focus on performance optimization, strengthening military readiness, resilience, and total well-being when they are equipped with basic nutrition principles.

#### Model Healthy Eating Behaviors

A good starting point to modeling healthy eating behaviors is for leaders to:

(1) Be educated in basic principles of nutrition and how those principles impact military performance. Encourage discussions about and around nutrition and eating.
(2) Know their resources: understand the roles of various healthcare providers, especially RDs, and how to refer SMs to those experts.
(3) Be willing to assess and analyze their food environment: standardized tools exist, including mNEAT.
(4) Have situational awareness: have SMs missed meals, been fueled by poor-quality foods, or not been given the opportunities to obtain healthy foods during their work hours? Are special occasions always marked with snack foods, desserts, and sugar-sweetened and/or alcoholic beverages?
(5) Know the resources aimed at evidence-based nutrition practices: digital (e.g., reputable websites, social media, and apps) and in-person options (health promotion/wellness departments, Armed Forces Wellness Centers).

Leaders should not impart their personal beliefs or practices on SMs, but instead consider the various backgrounds and practices of SMs and be aware that even if a particular way of eating works for them, it may not be appropriate for all SMs. LIN is not a framework in which the leader takes on the role of an RD; rather, leaders can model healthy practices within a team using basic evidence-based nutrition knowledge. Some examples include: leaders encouraging their team to follow the color coding of Go for Green^®^ [50] to select healthy “Green” choices more often in the dining facility, suggesting an SM with concerns about their body composition consult with an RD, or recognizing their team has not taken a moment to refuel and encouraging them to do so.

Leaders are not expected to provide calorie and/or macronutrient prescriptions or develop meal plans for their SM. The key to LIN as it applies to the leader is to encourage their SM to think about what they are eating, when they are eating, and why they are eating. Will the food choice selected by an SM help them capitalize on their performance for their particular job tasks (e.g., physical fitness tests, performance in weapons training and qualification, or sitting through a 3 h meeting)?

An engaged leader of LIN responsibly and proactively uses evidence-based information provided through credible sources such as the Uniformed Services University’s Consortium for Health and Military Performance (CHAMP) Human Performance Resources by CHAMP (HPRC) (hprc-online.org) and DoD health policy guidance to create a culture of health for their team. People often get nutrition information from sources that are not credible or evidence-based, such as social media, supplement vendors, fitness centers, non-peer-reviewed articles, and studies suffering from methodological biases, which leads to confusion and frustration [52,53]. Leaders should promote and encourage only credible resources.

Ultimately, leaders who endorse or adopt LIN principles will be more conscious of how they are fueling their own bodies, more informed about their health, and more likely to seek out experts, such as an RD, as needed. Leaders can also model healthy behaviors by taking time to enjoy unit camaraderie during mealtime and connect with their team. Overall, the intended outcome is that leaders are engaged in modeling positive behaviors using a top-down approach to improve nutritional fitness, dietary intake, and wellness.

### 2.4. Pillar 4: Consider Economic Factors

Economic access to food is another factor to consider when assessing the SM’s food security status [54]. As mentioned previously, lack of food security is an economic condition where a household does not have consistent, dependable access to enough food for active, healthy living [10]. The economic conditions that lead to food security concerns include both limited financial assets of the household, unemployment, and external shocks to the broader economy [55]. In a recent study of soldiers at a large Army base during the Coronavirus disease 2019 (COVID-19) pandemic, Rabbitt et al. found that soldiers who experienced concerns regarding food security were more likely to report financial insecurity and the lack of job security of their family members [56]. Studies have shown that households across the income spectrum can be food insecure [57], but households with liquid assets, like cash savings, are less likely to be food insecure. [58] At the same time, external constraints to a household’s food budget, like increased food prices, can lead to increased issues of food security as well [59]. Beyond food security, limited food access is an economic condition where households likely have little to no physical access to supermarkets, grocery stores, or other sources of healthy and affordable food in their community, and low incomes that make it financially challenging to travel elsewhere for nutritious and affordable foods [60].

There are resources to assist SMs in overcoming economic challenges to ensure they have adequate food. Federal nutrition assistance programs, like USDA’s Supplemental Nutrition Assistance Program (SNAP) and the Special Supplemental Nutrition Program for Women, Infants, and Children (WIC), are designed to improve food security and nutrition. Most households with gross incomes at or below 130% of the federal poverty line are eligible to participate in SNAP [61] while gross incomes at or below 185% of the poverty line are eligible to participate in WIC [62]. Leaders can also connect SMs to financial counselors and other Military OneSource resources that offer personal finance and savings advice [63]. Additionally, leaders can encourage SMs to utilize DoD commissaries as an accessible and affordable source of healthy foods to overcome the challenges of limited food access. Commissaries are located on most military installations and are strategically postured to serve SMs and their families. Leaders can also explore opportunities to connect with resources (e.g., RD, health promotion staff, commissary staff) who can use the commissary as a classroom to provide groups with tactile and in-the-moment experiences, such as food tastings and commissary tours, to reinforce nutrition education. Additionally, military installations, such as Joint Base Lewis-McChord, have established a food pantry on-site for staff; implementing food pantries could be an innovative leadership strategy to address food security by offering access to nutritious foods. When units are engaged in supporting one another, and resources are accessible to the SM, the risk of food insecurity may decrease [64].

### 2.5. Pillar 5: Promote Utilization of DoD and Partners’ Wellness Resources

Ample evidence-based resources are available for leaders to be aware of, leverage, and promote for optimal nutrition. For example, the Uniformed Services University, through CHAMP and its programs, contributes to mission readiness in the fields of human performance optimization (HPO) and TFF through education and training of the DoD workforce, their families, healthcare professionals, supporting agencies, and the public at large. HPRC [65] and Operation Supplement Safety (OPSS) [66] are the main vectors for education. HPRC serves as the public-facing website for disseminating evidence-based HPO resources, while OPSS is the public-facing website for evidence-based information on dietary supplements. Military One Source provides a wide variety of open-access resources to all military-connected individuals via an extensive website and call center. Nutrition, wellness, and food security are focus topics on Military One Source, thus making it a key platform for the education of leaders. In particular, Military One Source has leader-focused talking points to assist in identifying, discussing, and supporting food security and the Military Leaders Economic Security Toolkit to educate leaders on food security, housing availability, and economic well-being [63]. As outlined in the above pillars, additional local resources are available across a range of topics and delivery platforms.

#### Leveraging LIN as Part of Recruitment and Retention Programs

Currently, all military branches of service are concerned with building the future bench for recruitment and retention. Some investments into programs using various readiness frameworks are already promoting LIN concepts proposed in this article. Select examples are described below.

Army’s Future Soldier Preparatory Course (piloted August 2022) is a three-week program to minimize recruitment challenges by supporting candidates who fall short on academic (i.e., the Armed Services Vocational Aptitude Battery (ASVAB) test) and/or physical fitness requirements, due to excessive body weight or fitness level [67]. Thus far, the program has proven to be successful, with a 90% or greater retention of potential candidates by leveraging the course [67]. Similarly, the Navy’s Future Sailor Preparatory Course offers similar three-week support for physical and academic readiness, including ASVAB tutoring and holistic health education to meet Navy standards [68]. Both programs are examples of leveraging LIN concepts by providing instructor role models and nutritional resources.

Another wide-scale program the Army has adopted is Holistic Health and Fitness (H2F), implemented in October 2020 as an integrative model optimizing individual and unit readiness, injury reduction, wellness, and a quicker return to duty [32]. H2F encompasses five domains: physical, nutritional, mental, sleep, and spiritual readiness to help soldiers enhance lethality. Multiple tools and programs to support nutritional fitness are available to assess and improve the nutritional environment on installations (e.g., Go for Green^®^, mNEAT). The Army has heavily invested in this program, which is projected to improve readiness across an SM’s military career continuum. The Navy’s ShipShape Program is a weight management program [69] for active duty, reserve, beneficiaries, and government civilians, assisting with weight loss goals, including strategies for healthy behavioral modifications. The Marines’ High Intensity Tactical Training (HITT) Program is a combat-focused, comprehensive program emphasizing strength conditioning, operational effectiveness, and athleticism [70]. The Program implements evidence-based practices of strength and conditioning principles, provides specialized HITT instructors, and can be scaled to individual and unit physical fitness sessions. Lastly, a unique program that falls under the Department of the Air Force is the 2-year Space Force pilot program [71]. This program entails a holistic health approach to fitness for Space Force guardians, emphasizing the optimization of physical fitness, sleep, nutrition, stress reduction, avoidance of harmful substances and behaviors, and enhancing social connectedness. As shown in these programs, LIN can be applied at the individual, unit, and strategic levels to empower leaders to engage with their SMs, provide relevant and evidence-based resources and training, and serve as a role model to establish a culture of health for operational readiness.

### 2.6. Pillar 6: Evaluate TFF Impacts

Leaders can leverage and incorporate the TFF framework encompassing the environmental, behavioral, nutritional, physical, psychological, medical/dental, and spiritual domains as a strategy to evaluate their current food and nutrition environment and assess SM nutrition holistically for improving their unit’s culture of health. As a starting point, leaders can request that their unit’s food environment be evaluated by the DoD mNEAT, which measures food policy, food availability, and behavioral design for a variety of food service venues [48]. Additionally, questions posed throughout this paper could be leveraged within units to assess the leader and SM’s healthy eating behaviors and nutritional well-being over time.

## 3. Discussion

As an approach to nutritional fitness, LIN aims to create an environment where healthy eating is having protected time to fuel, equipping SMs with nutrition knowledge, and making SMs aware of available resources to optimize their health and performance.

Leaders can play a role in promoting a healthy nutrition environment, modeling top-down behaviors, and connecting SMs with evidence-based resources. With awareness of their community’s nutrition landscape, leaders can help support the nutrition programs available to best fuel performance and recovery among SMs.

The LIN framework is proposed at a critical time when the all-volunteer military force is facing a multitude of challenges, many of which have nutrition implications. First, food security has come to the forefront as a major challenge among active-duty personnel. The DoD released its first-ever food security strategy document in July 2022, Strengthening Food Security in the Force: Strategy and Roadmap, which describes six lines of effort to directly improve the food security status of active-duty members [14]. However, to be fully realized, many of the actions and initiatives described in the document need promotion at the unit level by local leaders and champions. Leaders can leverage LIN to help improve healthy food access among those in their chain of command.

LIN advances the current knowledge by strategically outlining how leaders can engage on nutrition readiness topics with SMs. Leaders using the LIN framework may help guide future DoD nutrition strategies from a policy and research perspective by addressing relevant areas identified within its seven core pillars. At present, there are no nutrition leadership (focused) frameworks within the military context that address the nutrition landscape specifically from a leader’s vantage point and provide a universal set of core values that extends to engaged leadership, community, trust, and role modeling.

LIN has the potential to assist the DoD in addressing some of the deficiencies identified in the 2022 and 2024 Government Accountability Office (GAO) reports [72,73]. Cited deficiencies included a lack of data on meal entitlement utilization and the need for establishing a nutrition leadership structure. Leaders adopting LIN are more likely to engage in a culture of health and to support meal entitlement utilization at government-funded dining facilities. By doing so, they can also drive economic and nutrition security, given that meals at these dining facilities meet nutrition specifications and are offered at discounted rates or as part of a meal entitlement. Finally, LIN can be leveraged as a framework to understand and categorize some of the nutrition-related issues addressed within the GAO report and can serve as a resource to leaders establishing a nutrition leadership structure as recommended within the GAO report.

The proposed LIN framework is limited by the lack of validation in the military population, but it offers a key opportunity for future research into nutrition leadership and SM readiness. Research could assess the entire LIN framework or its individual pillars within various DoD settings (academic departments, leadership courses, military treatment facilities, line units). Different military services may require tailored nutrition readiness education due to their distinct operational missions. However, quick wins from LIN, such as positive leader role modeling (healthy eating) and leveraging DoD-approved resources (CHAMP, OPSS), are readily available and can be integrated. In addition, while LIN was developed by experts within and outside the military nutrition environment, further stakeholder support and investment are required for implementing LIN from the strategic to the SM level. It is beyond the scope of the military leader (or any leader applying LIN as a framework) to diagnose deficiency or provide nutrition interventions. However, leaders can empower and equip their SMs with the appropriate resources and encourage healthy habits such as eating more fruits and vegetables, drinking water, cooking more, sharing a meal together, and reaching out to their local RD. Leaders are influential and can engage with their teams utilizing the right tools and messaging to combat threats to military readiness, including obesity and its associated chronic conditions, rising numbers of musculoskeletal injuries, and the increasing concern of food security within the ranks.

## 4. Conclusions

The role of the military leader is to empower SMs to be fit for duty. As a critical part of readiness and overall well-being, leaders should routinely engage with their SMs on nutritional fitness readiness and overall well-being. Leaders should also identify if they have policies that support a culture of health and nutrition readiness; if not, leaders within DoD organizations can encourage staff members in the development of a health and wellness council to address nutrition and nutrition environment policies. Additionally, a health and wellness council can be a powerful engine for an organization, especially when the council intentionally aligns its efforts with current organizational priorities and policies to bring together internal and external key stakeholders to increase SM and patron well-being. LIN offers a framework to build a culture of health to support military readiness. Leaders should be curious by asking open-ended questions and listening actively without judgment and value their personnel’s unique perspectives and challenges to nutritional fitness. Additionally, when leaders communicate with other leaders—both peers and superiors—they can affect nutrition-related changes through education and nutritional policy, both up the chain of command and down into the ranks.

## Data Availability

No new data were created or analyzed in this study. Data Sharing is not applicable to this article.
